# Editorial: Novel Therapeutic Target and Drug Development in Neurovascular Retinal Diseases

**DOI:** 10.3389/fphar.2021.657684

**Published:** 2021-04-15

**Authors:** Zhuo Shao, Zhongxiao Wang, Amy C. Y. Lo, Zhongjie Fu

**Affiliations:** ^1^ Division of Clinical and Metabolic Genetics, The Hospital for Sick Children, University of Toronto, Toronto, ON, Canada; ^2^ Department of Ophthalmology, Boston Children’s Hospital, Harvard Medical School, Boston, MA, United States; ^3^ Department of Ophthalmology, LKS Faculty of Medicine, The University of Hong Kong, Hong Kong, China

**Keywords:** neovascularization, retina, retinopathy, metabolism, epigenetic modification, microfluidics, drug discovery, small peptide

**Editorial on the Research Topic** **Novel Therapeutic Target and Drug Development in Neurovascular Retinal Diseases**Pathological ocular angiogenesis leads to blindness in retinopathy of prematurity (ROP), diabetic retinopathy (DR), and age-related macular degeneration (AMD). Clinically approved anti-VEGF therapy has limited effectiveness and side effect profile unfit for some patients (Bressler et al., 2012; Jalali et al., 2013; Klufas and Chan, 2015; Zhao and Singh, 2018; Bakri et al., 2019). Therefore, further understanding of the disease pathogenesis and exploration of new therapeutics are required. In this research topic, we highlighted new drug targets, therapeutic approaches, and technologies for treatment of ocular neovascularization.

## Metabolism and Cell-Cell Interaction

Understanding the interaction of endothelial cells with the surrounding cells is essential for the development of effective and safe therapeutics (Wilson and Sapieha, 2016; Binet et al., 2020; Fu et al., 2020). Neuronal metabolism regulates retinal vascular function (Joyal et al., 2018; Fu et al., 2019). In this research topic, Fouda et al. provided a systematic overview of the arginase pathway in acute retina and brain injury, and discussed the possibility of modulating this pathway to treat ischemia-induced neurodegeneration. Shetty and Corson summarized the vulnerability of endothelial cells to mitochondrial heme loss, and proposed that targeting intracellular heme via inhibiting heme synthesis or blocking heme transport may be a novel strategy to decrease retinal neovascularization. Further exploration of neural-vascular metabolism and interaction is needed. Endothelial cells utilize glucose, fatty acid and glutamine as substrates for energy and biomass for cell homeostasis and growth (Falkenberg et al., 2019). On the other hand, photoreceptors require glucose and fatty acids for energy production and function (Joyal et al., 2016). Therefore, when considering interventions for metabolic modulation, it is necessary to take into account the overall impact on various retinal cell types.

In addition, the interaction of metabolic pathways in retinopathies also requires further investigation. Recently, low serine with increase in deoxysphingolipids is reported to correlate with macular disease (Gantner et al., 2019). Wang et al. revealed significant metabolic disturbances (such as amino acids and ketone bodies) in aqueous humor of patients with Posner-Schlossman syndrome that were identified with metabolomics. Further exploration of retinal metabolic interactions between amino acid, lipid pathways, and others would definitely attract great interests.

Inflammation and autophagy are induced in response to stressed conditions such as in retinal metabolic disorders (Tang and Kern, 2011; Mitter et al., 2012; Kauppinen et al., 2016). Wang et al. discussed that persistent neuroinflammation exacerbates ocular neovascularization. They further explored the potential involvement of SOCS3 and c-Fos in the disease pathogenesis of retinopathies. Zhu et al. demonstrated that rapamycin induced autophagy and preserved trabecular meshwork cells in glucocorticoid-induced glaucoma mice can be a potential therapeutic approach to glaucoma.

## Genomics, Transcriptome, and Proteomics for Drug Target Identification

Recently, genomic analysis, transcriptome profiling, and proteomics have been used as a hypothesis free approach to identify drug targets in retinal neovascular diseases (Vahatupa et al., 2018; Desjarlais et al., 2019). Desjarlais et al. reported the discovery of down regulation of MicroRNA-96 in oxygen induced retinopathy (OIR) rats through next generation sequencing (NGS) screening. *In vitro* study demonstrated that overexpression of MicroRNA-96 stimulated tubulogenesis and migration against hyperoxia-induced endothelial dysfunction, while antagonizing microRNA-96 led to angiogenic impairment. Intravitreally supplementing microRNA-96 mimic preserved retinal/choroidal microvessels in the hyperoxic state of rat OIR model. Cheng et al. analyzed and compared transcriptome profiles in retinal-choroid tissues derived from donor patients with AMD and healthy controls. They identified that *EFEMP1* gene was upregulated in the AMD, especially wet-AMD patients. Elevation of *EFEMP1* product, fibulin-3, was confirmed in the serum of wet-AMD patients. *In vitro* overexpression and knockdown of *EFEMP1* in human umbilical vein endothelial cells (HUVECs) confirmed the proangiogenic effect of this gene. Vähätupa et al. reviewed the elevation of crystallins, small heat shock proteins, during early hypoxic state of OIR as well as an increase of actomyosin complex and Filamin A-R-Ras axis at the peak of neovascularization that were discovered through proteomic analysis using sequential window acquisition of all theoretical mass spectra (SWATH-MS). Some crystallins are neuroprotective while others play a prominent role in the pathology of neovascularization. The actomyosin complex and Filamin A-R-Ras axis regulates vascular permeability of the angiogenic blood vessels. These proteomic changes were also confirmed patients with proliferative diabetic retinopathy (PDR) and retinal vein occlusion (RVO).

## RNA and Peptide Based Therapeutic Approaches

RNA based therapeutic approaches against retinal neovascular disease have gained significant interest in recent years. Ma et al. found that silencing *Trpc6* with RNA interference (RNAi) abolished high glucose-induced decreases in glutamate uptake and Müller glial cell death *in vitro*, suggesting that TRPC6 may be a promising target that deserves further investigation in animal models. Protection of neurovascular supporting cells Müller glia and regulation of Müller gliosis may protect against diabetic retinopathy (Coughlin et al., 2017; Le, 2017). Additionally, Guan et al. reported that MicroRNA-18a-5p is increased during neovascularization of OIR mice retina, adding to the list of miRNAs that is involved in this process (Zhou et al., 2016; Xia et al., 2018). Antagonizing MicroRNA-18a-5p using agomiR-18a-5p suppressed neovascularization in OIR mice and Human Retinal Microvascular Endothelial Cell (HRMEC) proliferation, migration, and tube formation. miRNA mimics or inhibitors have been tested in clinical trials for treatment of viral infection (Janssen et al., 2013) and malignancy (Van Zandwijk et al., 2017). Moreover, long none coding RNAs (lncRNAs) have been shown to participate in transcription, post-transcription, translation, epigenetic regulation, splicing, and intracellular/extracellular trafficking (Wilusz et al., 2009). Gong et al. characterized the role of lncRNA human testis development–related gene 1 (TDRG1) in proliferative DR through modulation of VEGF. LncRNA TDRG1 was elevated in fibrovascular membranes (FVMs) from DR patients and hyperglycemic treated HRMECs. Knockdown of lncRNA TDRG1 reduced the VEGF level in HRMECs and protect against high-glucose-stimulated HRMEC migration.

In the area of peptide-based therapies, Sun et al. tested a small peptide derived from human tissue-type plasminogen kringle 2 (t-PA kringle 2) in HRMEC and mice model of OIR. Their study demonstrated that this peptide effectively inhibits HRMEC proliferation, migration and tube formation. It also inhibited retinal neovascularization in OIR mice retina. Compared to proteins, small peptides present the advantage of easier and relatively inexpensive synthesis, higher consistency between batches, lower immunogenicity, higher solubility in water, and better penetrating abilities. On the other hand, Ibuki et al. focused on Lactoferrin, a type of glycoprotein that is naturally present in body fluids. In this study, oral administration of Lactoferrin was shown to reduce laser-induced CNV in mice through inhibition of Hypoxia Induced Factor (HIF). Similarly, lactoferrin was shown to inhibit HIF in the 661W cone photoreceptor cell line.

## Advanced Technologies

2D cell culture model has been widely used for mechanistic investigations and drug tests. However, this approach is limited by altered extracellular microenvironment, cell morphology and polarity, as well as nutrition depletion and waste product accumulation in media (Kapalczynska et al., 2018). Bai and Wang summarized 3D organoid and microfluidic system as tools for the study of organ function and ophthalmic drug delivery. Bai et al. also reported the detailed methodology on the development of cornea-on-a-chip using primary murine corneal epithelial and endothelial cells. Taking the advantage of better modeling *in vivo* conditions, 3D culture is believed to improve the disease pathogenesis study and drug testing process.

In conclusion, this research topic includes original studies and reviews regarding novel therapeutic approaches to neurovascular retinopathies. Improved understanding of the metabolic and inflammatory aspects of cell-cell interaction expands potential drug target for retinal neovascularization. Novel therapeutic approaches using RNA and peptide-based molecules diversifies the therapeutic approaches to these blinding diseases, where newly developed models such as 3D culture holds the promises of expediting and reducing the cost of drug discovery process. As Topic Editors of this special issue, we sincerely thank all the authors and reviewers for their valuable contributions to this research topic.
